# Functional analysis of novel A20 variants in patients with atypical inflammatory diseases

**DOI:** 10.1186/s13075-021-02434-w

**Published:** 2021-02-06

**Authors:** Saori Kadowaki, Kunio Hashimoto, Toyoki Nishimura, Kenichi Kashimada, Tomonori Kadowaki, Norio Kawamoto, Kohsuke Imai, Satoshi Okada, Hirokazu Kanegane, Hidenori Ohnishi

**Affiliations:** 1 Department of Pediatrics, Gifu University Graduate School of Medicine, 1-1 Yanagido, Gifu, Gifu 501-1194 Japan; 2 Department of Pediatrics, Nagasaki University Graduate School of Biomedical Sciences, Nagasaki, Japan; 3 Division of Pediatrics, Developmental and Urological-Reproductive Medicine Faculty of Medicine, University of Miyazaki, Miyazaki, Japan; 4 Department of Pediatrics and Developmental Biology, Tokyo Medical and Dental University (TMDU), Tokyo, Japan; 5 Department of Pediatrics, National Hospital Organization, Nagara Medical Center, Gifu, Japan; 6 Department of Community Pediatrics, Perinatal and Maternal Medicine, Graduate School of Medical and Dental Sciences, Tokyo Medical and Dental University (TMDU), Tokyo, Japan; 7 Department of Pediatrics, Hiroshima University Graduate School of Biomedical and Health Sciences, Hiroshima, Japan; 8 Department of Child Health and Development, Graduate School of Medical and Dental Sciences, Tokyo Medical and Dental University (TMDU), Tokyo, Japan; 9 Clinical Genetics Center, Gifu University Hospital, Gifu, Japan

**Keywords:** A20 haploinsufficiency, In vitro assay, Missense variant, *TNFAIP3* gene, Variant classification

## Abstract

**Background:**

A20 haploinsufficiency (HA20) is an early-onset autoinflammatory disease caused by mutations in the *TNFAIP3* gene, which encodes the protein A20. Numerous truncating mutations in the *TNFAIP3* gene have been reported in HA20 patients, whereas fewer missense variants have had their pathogenicity confirmed. Here, we evaluated the pathogenic significance of three previously unreported missense variants of the *TNFAIP3* gene in suspected cases of HA20.

**Methods:**

We obtained the clinical features and immunological data of three patients with missense variants (Glu192Lys, Ile310Thr, and Gln709Arg) of unknown significance of *TNFAIP3*. We then performed in vitro functional assays including analysis of nuclear factor (NF)-κB reporter gene activity, detection of A20 expression and phosphorylation of A20 by IκB kinase β (IKKβ), and K63-deubiquitination assay using *TNFAIP3*-deficient HEK293 cells. Three known pathogenic missense mutations reported previously were also investigated.

**Results:**

The inhibitory effect on NF-κB reporter gene activity was significantly disrupted by A20 Glu192Lys and the three known mutations. The variants Ile310Thr and Gln709Arg did not show a difference from the wild type in any of the assays performed in this study.

**Conclusions:**

Among the three variants in the *TNFAIP3* gene, Glu192Lys was interpreted as being likely pathogenic, but Ile310Thr and Gln709Arg as being not pathogenic (uncertain significance and likely benign, respectively), based on the American College of Medical Genetics and Genomics standards and guidelines. Our study highlights the necessity of performing in vitro functional assays, notably, NF-κB reporter gene assay, to evaluate the pathogenicity of *TNFAIP3* missense variants for the accurate diagnosis of HA20.

**Supplementary Information:**

The online version contains supplementary material available at 10.1186/s13075-021-02434-w.

## Background

A20 haploinsufficiency (HA20) is an early-onset autoinflammatory disease caused by mutations in the *TNFAIP3* gene. The major phenotype of HA20 is Behçet’s disease (BD)-like symptoms, including recurrent aphthous stomatitis, genital ulcers, and intestinal symptoms, but clinical features show diversity, including not only BD-like symptoms but also autoinflammatory and/or autoimmune symptoms. Furthermore, some atypical cases with HA20 have been reported, such as late-onset symptoms or autoimmune lymphoproliferative syndrome (ALPS)-like phenotype with multiple autoimmune diseases [1]. A20, the protein encoded by the *TNFAIP3* gene, suppresses the interactions of tumor necrosis factor (TNF), IL-1 family, toll-like receptor (TLR), B-cell receptor (BCR), and T-cell receptor (TCR) with the NF-κB signaling pathway. Numerous mutations in the *TNFAIP3* gene have been reported in HA20 patients since the first report on them in 2016 [2], most of which have truncating effects due to nonsense, frameshift, and splice site mutations [3]. Compared with the number of truncating mutations, few missense variants have been registered as pathogenic or likely pathogenic in the Infevers database (https://infevers.umai-montpellier.fr/web/index.php). Recently, guidelines for the genetic diagnosis of monogenic autoinflammatory diseases, including those involving the *TNFAIP3* gene, were presented [4], which suggested that in vitro functional assays are needed to correctly interpret the clinical significance of the variants, especially regarding nonsynonymous variants showing unknown pathogenicity.

In our previous study, the *TNFAIP3* missense mutation c.728G>A (p.Cys243Tyr) showed no significant difference from the wild type (WT) in the evaluation of NF-κB reporter gene activation by TNF-α stimulation, although its protein expression level was reduced [5]. Biallelic somatic loss-of-function mutations of *TNFAIP3* drive the onset of malignant lymphoma due to the excess activation of NF-κB signaling, and the associated problem of interpreting the effects of missense mutations of *TNFAIP3* is similar to that on the onset of HA20. Against this background, it was reported that some *TNFAIP3* missense variants identified in tumors could show significant differences from the WT in the NF-κB reporter gene activity induced by co-transfection of gain-of-function (GoF) mutations of *MYD88* or caspase recruitment domain family member 11 (*CARD11*), which activate TLR or BCR signaling [6]. Furthermore, the functional significance of single-nucleotide substitution, such as c.380T>G (p.Phe127Cys; rs2230926), was evaluated by controlling the expression level of A20 using A20-deficient cells [7].

In this study, we identified three different previously unreported missense variants of the *TNFAIP3* gene in suspected cases of HA20 including an atypical but possible case as mentioned above, and designed an in vitro assay combining the methods described in the literature to perform a multi-faceted evaluation of their pathogenicity. Furthermore, using the obtained results, we classified each variant based on the American College of Medical Genetics and Genomics (ACMG) standards and guidelines [8].

## Methods

### Patients

Patient 1 was a 4-year-old boy. At 11 months old, he had been hospitalized for cervical lymphadenitis and then suffered from repeated tonsillitis, cervical lymphadenitis, and bronchitis with elevated serum C-reactive protein (CRP). He was initially suspected of having an immunodeficiency disease but had no abnormalities in neutrophil function, lymphocyte fraction, or immunoglobulin levels. During the fever episodes, no significant pathogens were detected and his fever often improved without antibiotics. Because a single dose of prednisolone (PSL) was effective for the fever, he was diagnosed with periodic fever, aphthous stomatitis, pharyngitis, and adenitis (PFAPA) syndrome based on Thomas’s criteria [9]. At the age of 4 years and 2 months, fever with pharyngeal redness persisted for 9 days, with PSL not being effective for it. The patient also had temporary nonbacterial coxitis. He had a heterozygous c.574G>A (p.Glu192Lys) variant of *TNFAIP3.* After undergoing tonsillectomy, the frequency of the fever episodes decreased. His father also suffered from recurrent stomatitis, folliculitis, and hemorrhoids and possessed the same variant of *TNFAIP3*.

Patient 2 was a 20-year-old woman. From around the age of 12, she had occasionally suffered from severe abdominal pain of unknown cause. Around 13 years of age, she had suffered from a recurrent fever that persisted for 3–7 days and abdominal pain with a high level of serum CRP approximately every 2 months. Serum inflammatory reactants were negative during the intermittent episodes of fever. Lower gastrointestinal endoscopy showed erosion in Peyer’s patch of the ileum. Histopathologically, this lesion showed ulcers and granulation tissue with inflammatory cell infiltration. This case was initially considered to possibly involve familial Mediterranean fever (FMF) related to the heterozygous c.329T>C (p.Leu110Pro; rs11466018) and c.442G>C (p.Glu148Gln; rs3743930) variants of *MEFV*. However, colchicine given for diagnostic and therapeutic purposes failed to reduce the fever attacks, while NSAIDs and PSL were also ineffective for the fever and abdominal pain. When infliximab treatment was started with methotrexate as secondary prevention, the episodes of fever and abdominal pain completely disappeared. Finally, the heterozygous c.929T>C (p.Ile310Thr; rs530310566) variant of *TNFAIP3* was identified in this patient. Her mother had the same variant but lacked any symptoms*.*

Patient 3 was a 24-year-old woman. She had suffered from athetoid cerebral palsy due to cardiopulmonary arrest of unknown cause on postnatal day 1. She had developed epilepsy at the age of 4 years and type 1 diabetes with positive anti-glutamic acid decarboxylase antibody at the age of 12 years. At the age of 24 years, she had a fever, facial erythema, and proteinuria. She was diagnosed with systemic lupus erythematosus (SLE) with the following findings: positive antinuclear antibodies, anti-dsDNA antibodies, anti-Sm antibodies, anti-cardiolipin antibodies, hypocomplementemia, and positive direct Coombs test. She had a heterozygous c.2126A>G (p.Gln709Arg) variant of *TNFAIP3*. Her father had the same variant but lacked any symptoms*.*

The variant Glu192Lys was identified among 55 patients suspected of having autoinflammatory diseases analyzed at Gifu University Hospital from October 2017 to September 2020. The patients with variants Ile310Thr and Gln709Arg were recruited by personal communication with the collaborators (Nagasaki University and Tokyo Medical and Dental University).

Written informed consent to participate in this study was obtained from each of the patients or their parents. This study was conducted in accordance with the Declaration of Helsinki and approved by the ethics boards of Gifu University and Tokyo Medical and Dental University.

### Genetic analysis

Target gene sequencing (TGS) for a primary immunodeficiency-related 400-gene panel was performed using genomic DNA from the whole blood of patients 1 and 2. Whole-exome sequencing (WES) was performed using genomic DNA from the whole blood of patient 3 and her parents. To confirm the results, Sanger sequencing was performed for the patients and their parents.

### Multicolor flow cytometry analysis

Peripheral blood samples from patients with *TNFAIP3* variants were analyzed by multicolor flow cytometry using a BD LSRFortessa flow cytometer (Becton Dickinson, Franklin Lakes, NJ). Patients’ peripheral blood mononuclear cells (PBMCs) were isolated from whole blood using Lymphoprep (Axis-Shield Diagnostics Ltd., Dundee, UK) gradient centrifugation, the details of which are described in our previous report [3].

### Variant frequency and in silico analysis

The frequencies of variants were obtained with reference to the Genome Aggregation Database (gnomAD) dataset v.2.1.1 (https://gnomad.broadinstitute.org) and Tohoku Medical Megabank Organization (ToMMo) 8.3KJPN v20200831 (https://jmorp.megabank.tohoku.ac.jp/202008/variants). The *TNFAIP3* variants p.Glu192Lys, p.Ile310Thr, and p.Gln709Arg were evaluated in silico using Polyphen-2 (http://genetics.bwh.harvard.edu/pph2/), Sorting Intolerant from Tolerant (SIFT, https://sift.bii.a-star.edu.sg), and Combined Annotation-Dependent Depletion (CADD, https://cadd.gs.washington.edu/snv).

### Cell culture

Human embryonic kidney (HEK)293 cells (purchased from the Japanese Collection of Research Bioresources, Osaka, Japan) were cultured in high-glucose Dulbecco’s modified Eagle’s medium (Invitrogen, Carlsbad, CA) supplemented with 10% heat-inactivated FCS (Sigma-Aldrich, St. Louis, MO), penicillin (100 U/mL), and streptomycin (100 μg/mL). A20-deficient HEK293 cells were created by the CRISPR/Cas9 system. The target sequence 5′-CAGTTTCGGGAGATCATCCA-3′ of exon 2 of the *TNFAIP3* gene was cleaved using pX330-U6-Chimeric_BB-CBh-hSpCas9 (Addgene Plasmid #42230). Successful disruption of the *TNFAIP3* gene was confirmed by the detection of DNA fragments of the target site by polymerase chain reaction and the direct sequencing of genomic DNA from candidate clones. A20-deficient cells were cultured in the same medium as mentioned above.

### Vector preparation

The cDNA encoding human *TNFAIP3* tagged at the N-terminus with a c-myc epitope (myc-*TNFAIP3*) was cloned into plasmid pcDNA3.1+ (Invitrogen). *TNFAIP3* missense or deletion variants, including c.380T>G (p.Phe127Cys; rs2230926), c.1245_1248del4 (p.Lys417Serfs*4, [1]), c.728G>A (p.Cys243Tyr, [10]), c.1428G>A (p.Met476Ile, [11]), c.1939A>C (p.Thr647Pro; rs142253225, [12]), c.574G>A (p.Glu192Lys), c.929T>C (p.Ile310Thr; rs530310566), c.2126A>G (p.Gln709Arg), c.2127A>C (p.Gln709H; genomic mutation ID: COSV52803693), and c.2125_2127del (p.Gln709del), were generated using the combination of KOD FX Neo and KOD Plus Mutagenesis Kit (Toyobo Co., Osaka, Japan), in accordance with the manufacturer’s instructions. Phe127Cys and Lys417Serfs*4 were a representative negative control as a non-pathogenic single-nucleotide polymorphism (SNP) and a representative positive control of a truncating mutation in this study, respectively. pcDNA3.1 *CARMA1*(*CARD11*) (plasmid #44431) was purchased from Addgene (Watertown, MA) and the Phe130Val mutation was generated. The cDNA encoding human *MYD88* was cloned into plasmid pAcGFP1-C1 (Clontech Laboratories, Mountain View, CA), and Leu265Pro mutation was also generated. The NF-κB luciferase reporter vector (pGL4.32-luc2P/NF-kappaB-RE/Hygro) and the *Renilla* luciferase reporter vector (pGL4.74-hRluc/TK) were purchased from Promega (Fitchburg, WI). The inducible expression vector (pcDNA4/TO) and the expression vector of the tetracycline (Tet) repressor (pcDNA6/TR) were purchased from Invitrogen. The cDNA encoding human *TNFAIP3* variants tagged at the N-terminus with a c-myc epitope was cloned into plasmid pcDNA4/TO. The vector expressing IKKβ tagged at the C-terminus with a FLAG epitope, pRK7 IKK2/IKKb (plasmid #26203), was purchased from Addgene. The cDNA encoding human *TNF receptor-associated factor 6* (*TRAF6*) tagged at the N-terminus with a FLAG epitope was cloned into plasmid pcDNA3.1+ (Invitrogen). The pRK5-HA-Ubiquitin-K63 (plasmid #17606) was purchased from Addgene; the HA tag was replaced with the V5 tag using the above-mentioned mutagenesis system.

### NF-κB reporter gene activity

A20-deficient HEK293 cells were transfected with 10 ng per well of pcDNA3.1+ mock vector or pcDNA3.1+ myc-A20 WT or variants in 96-well plates using Lipofectamine 2000 (Invitrogen), in accordance with the manufacturer’s instructions. The NF-κB luciferase reporter and *Renilla* luciferase reporter vectors were co-transfected. Three experimental conditions were applied, involving the following different stimulations: co-transfection with *CARD11* GoF mutant or *MYD88* GoF mutant, and TNF-α stimulation. Two groups were co-transfected with *CARD11* Phe130Val or *MYD88* Leu265Pro. After transfection, cells were incubated for 24 h, and then the third group without co-transfection of these GoF mutants was stimulated with 20 ng/mL TNF-α (R&D, Minneapolis, MN) for 6 h. Luciferase reporter activity was analyzed using the Dual-Luciferase Reporter Assay System (Promega). Values are expressed as a mean of three independent experiments with technical triplicates ± SD. The statistical significance of differences in luciferase activity compared with WT stimulated with TNF-α or *MYD88* or *CARD11* GoF mutant was analyzed using one-way ANOVA with Tukey’s multiple comparisons test. *P* values of less than .05 were considered statistically significant. *P* values of less than .05, less than .01, less than .001, and less than .0001 are indicated with *, **, ***, and ****, respectively.

### Immunoblot analysis

To detect the protein expression, HEK293 cells were transfected with a pcDNA3.1+ control vector or pcDNA3.1+ myc-A20 WT or variants using Lipofectamine 2000, in accordance with the manufacturer’s instructions. After 48 h of incubation, cells were harvested and lysed. All extracts were adjusted to contain equal amounts of total cellular proteins. The whole-cell lysates were analyzed using western blotting. The samples were separated by electrophoresis on sodium dodecyl sulfate polyacrylamide gels and transferred to PVDF membranes using an iBlot 2 Dry Blotting System (Invitrogen). A20 and β-actin proteins were detected with an anti-myc antibody (Invitrogen) and anti-β-actin antibody (Sigma-Aldrich), followed by incubation with an anti-mouse IgG horseradish peroxidase conjugate (Promega).

### Nuclear factor-κB reporter gene activity of the dose-dependent A20 expression using the Tet-on system

A20-deficient HEK293 cells were transfected with 10 ng per well of pcDNA4/TO+ mock vector or pcDNA4/TO+ myc-A20 WT or variants in 96-well plates using Lipofectamine 2000 (Invitrogen). The pcDNA6/TR plasmid (six times as much as the A20 expression vector), the NF-κB luciferase reporter, and *Renilla* luciferase reporter vectors with pAcGFP1-C1-*MYD88* Leu265Pro were co-transfected. After transfection, cells were incubated for 24 h, the medium was removed, and fresh medium containing tetracycline was added to the cells. The final concentrations of tetracycline were 0, 10, 25, 50, and 100 ng/mL. After 24 h of incubation, luciferase reporter activity was analyzed using the Dual-Luciferase Reporter Assay System. The activities of WT and each variant were normalized to that of mock incubated with 0 ng/mL tetracycline. Values are expressed as the mean of three independent experiments with technical triplicates ± SD.

### Evaluation of IKKβ-mediated A20 phosphorylation

To evaluate IKKβ-mediated phosphorylation of the A20 serine residue (S381), A20-deficient HEK293 cells were seeded in six-well plates at a density of 4.0 × 10^5^/mL and transfected with 0.5 μg of plasmids expressing IKKβ, together with 1.5 μg of myc-tagged *TNFAIP3* WT or variants. Cells were harvested 48 h after transfection. All extracts were adjusted to contain equal amounts of total cellular proteins, as determined using the Bradford method. The whole-cell lysates and supernatants of cell lysates were analyzed. Phosphorylated A20 was detected with anti-phospho-A20/TNFAIP3 (Ser381) antibody (Cell Signaling Technology, Danvers, MA), followed by incubation with anti-rabbit IgG horseradish peroxidase conjugate (Promega). IKKβ was detected with anti-FLAG antibody (Sigma-Aldrich).

### Deubiquitination assay

For the deubiquitination assay of K63-linked polyubiquitin chains, A20-deficient HEK293 cells were seeded in six-well plates at a density of 4.0 × 10^5^/mL and transfected with 0.75 μg of plasmids expressing target protein (human TRAF6), together with 0.75 μg of pRK5-V5-Ubiquitin-K63 and 1.0 μg of myc-tagged *TNFAIP3* WT or variants. Cells were harvested 48 h after transfection. All extracts were adjusted to contain equal amounts of total cellular proteins, as determined using the Bradford method. The supernatants of cell lysates were subjected to immunoprecipitation with anti-FLAG antibodies (Sigma-Aldrich) against TRAF6 and subsequently blotted with anti-V5 antibodies (Medical & Biological Laboratories Co., Nagoya, Japan) against K63-linked ubiquitin.

## Results

**Fig. 1:**
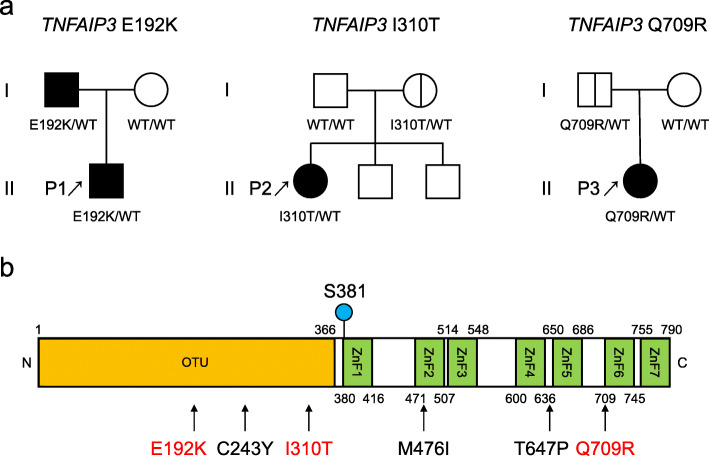
**a** Pedigree of the families with heterozygous *TNFAIP3* variants. **b** The locations of the missense variants on the domain structure of the A20 protein. The panel includes the previously reported missense mutations (C243Y, M476I, and T647P) and the three variants evaluated in this study

The characteristics of the patients with the *TNFAIP3* variant and the pedigree of the families are shown in Table 1 and Fig. 1a. Figure 1b shows the locations of the missense variants on the domain structure of A20 protein, including the previously reported missense mutations and the three variants evaluated in this study. Supplementary Table S1 shows the results of the multicolor flow cytometry of the patients. Patient 1 had been treated with cimetidine while patient 2 had been treated with infliximab at the time of analysis. Patient 3 had been treated with PSL, taltirelin hydrate, anti-epileptic drugs, and insulin at the time of analysis. The levels of double-negative T cells, regulatory T cells, T helper 17 cells, and follicular helper T cells of the present patients tended to increase compared with those of age-matched control groups, as previously reported [13].

**Table 1:** Characteristics of patients with *TNFAIP3* missense variants

	Patient no.		
	P1	P2	P3
Sex	Male	Female	Female
Age of onset	11 m	12 y	12 y
Age of analysis	4 y	20 y	24 y
*TNFAIP3* variant	c.574G>A	c.929T>C	c.2126A>G
	p.Glu192Lys	p.Ile310Thr	p.Gln709Arg
Clinical symptom	Recurrent fever, pharyngitis, coxitis	Recurrent fever, abdominal pain	Fever, facial erythema, proteinuria
Autoantibody	No	No	ANA, anti-dsDNA, anti-Sm, anti-CL, anti-GAD antibody
The diagnosis before the identification of *TNFAIP3* variants	PFAPA	Colchicine-resistant FMF (infliximab effective), erosion of the ileum	Type 1 diabetes, SLE
With the same variant in the family	Father (recurrent stomatitis, hemorrhoids, folliculitis)	Mother (asymptomatic)	Father (asymptomatic)

*m* months, *y* years, *ANA* antinuclear antibody, *anti-CL antibody* anti-cardiolipin antibody, *anti-GAD antibody* anti-glutamic acid decarboxylase antibody, *PFAPA* periodic fever, aphthous stomatitis, pharyngitis, and adenitis, *FMF* familial Mediterranean fever, *SLE* systemic lupus erythematosus

The prevalence rates of the *TNFAIP3* variants Glu192Lys and Gln709Arg were not registered in the gnomAD v2.1.1 database or ToMMo 8.3KJPN. The prevalence of the Ile310Thr allele was 0.0002 in Europe and 0.0 in East Asians from the gnomAD, and 0.0001 from ToMMo 8.3KJPN. The sequence alignment evaluated by Basic Local Alignment Search Tool (BLAST, https://blast.ncbi.nlm.nih.gov/Blast.cgi#) showed that Glu192 is located at a highly conserved region and Ile310 and Gln709 are located at partially conserved regions (Supplementary Table S2). Regarding Glu192Lys, in silico analysis identified this variant as probably damaging with a score of 0.999 in Polyphen-2 and 32 in CADD (Table 2 and Supplementary Fig. S1). However, for Ile310Thr, there were conflicting results of the three in silico prediction methods, with it being identified as either damaging or benign. Meanwhile, Gln709Arg was evaluated as tolerated or benign.

**Table 2:** Summary of three *TNFAIP3* variants (results of analysis and the variant classification)

	*TNFAIP3* variant		
	p.Glu192Lys	p.Ile310Thr	p.Gln709Arg
Frequency in Europe^a^	NA	0.00002	NA
Frequency in East Asia^a^	NA	0	NA
Frequency in Japan^b^	NA	0.0001	NA
Polyphen-2 score	0.999	0.063	0.046
SIFT score	NA	0.05	0.68
CADD score	32	22.6	11.04
NF-κB reporter gene activity^c^	Not suppressed	Suppressed	Suppressed
Expression of protein^c^	Not decreased	Mildly decreased	Not decreased
A20 Ser381 phosphorylation^c^	Not decreased	Not decreased	Not decreased
Deubiquitinase function^c^	Retained	Retained	Retained
Variant classification	Likely pathogenic	Uncertain significance	Likely benign

*NA* not available. ^a^Information of allele frequency was referred from gnomeAD v2.1.1. ^b^Information of allele frequency was referred from ToMMo 8.3KJPN. ^c^Results were evaluated based on comparison with wild type

**Fig. 2:**
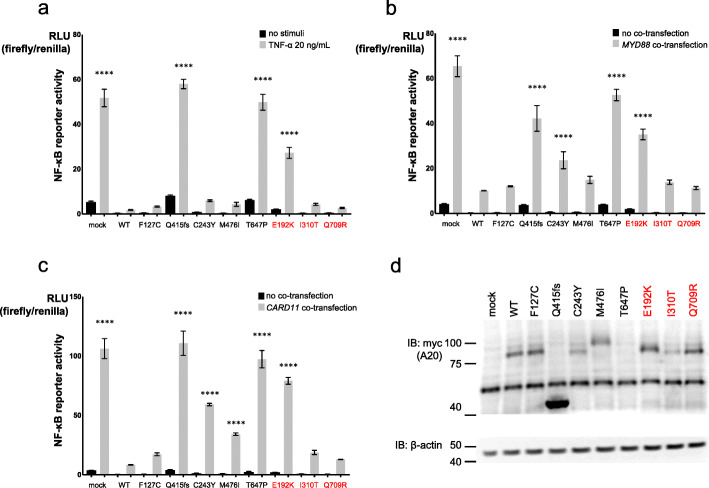
NF-κB reporter gene activity of *TNFAIP3* variants and the expression of A20. **a**–**c** NF-κB reporter gene analysis measuring activity of *TNFAIP3* variants using A20-deficient HEK293 cells. NF-κB reporter gene activity was induced by **a** stimulation with 20 ng/mL TNF-α, **b** co-transfection with *MYD88* mutant (L265P), and **c** co-transfection with *CARD11* mutant (F130V). The variant E192K could not suppress the NF-κB reporter gene activity elevated by TNF-α stimulation or co-transfection of GoF mutants of *MYD88* or *CARD11*. **d** Immunoblot analysis of A20 variants. The expression levels of the variant I310T and known mutants, C243Y and T647P, were reduced

The inhibitory effect on NF-κB reporter gene activity was significantly disrupted by *TNFAIP3* Glu192Lys compared with that of WT upon stimulation with TNF-α or co-transfection of *MYD88* and *CARD11* (Fig. 2a–c). The inhibitory effect on NF-κB reporter gene activity was also significantly disrupted by *TNFAIP3* missense variants Cys243Tyr, Met476Ile, and Thr647Pro, which were previously reported, upon stimulation with co-transfection of *MYD88* or *CARD11* GoF mutant. However, the *TNFAIP3* variants Ile310Thr and Gln709Arg suppressed the elevation of NF-κB reporter gene activity, similarly to the WT and Phe127Cys.

The levels of protein expression for myc-tagged *TNFAIP3* variants Glu192Lys and Gln709Arg were similar to the WT expression levels, while that of Ile310Thr was mildly decreased but not abolished. Among the previously reported missense variants, the protein expression level of Cys243Tyr was mildly reduced and that of Thr647Pro was reduced compared with those of the WT (Fig. 2d).

**Fig. 3:**
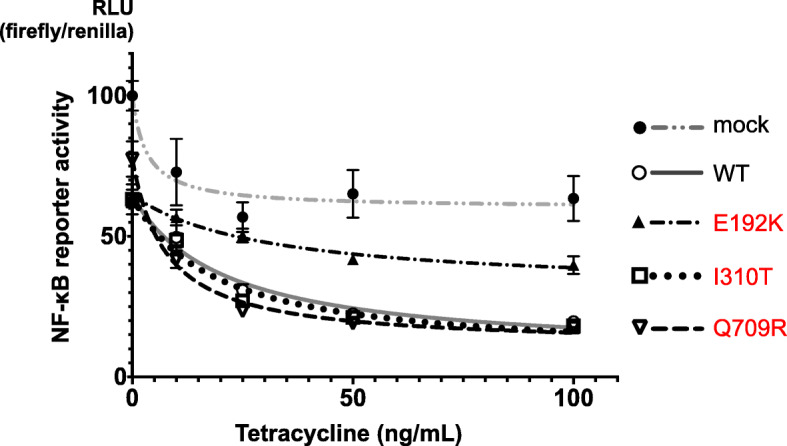
NF-κB reporter gene activity of *TNFAIP3* variants using A20-deficient HEK293 cells and the Tet-on system. The expression levels of A20 were controlled by the Tet-on system. The variant E192K could not suppress the increase of activity regardless of the A20 expression level compared with the WT

Evaluation of NF-κB reporter gene activity using the Tet-on system showed that *TNFAIP3* WT suppressed this activity as the expression level of A20 increased (Fig. 3). The variant Glu192Lys could not suppress the increase of activity regardless of the A20 expression level, in contrast to the WT, while the variants Ile310Thr and Gln709Arg suppressed it as well as the WT.

**Fig. 4:**
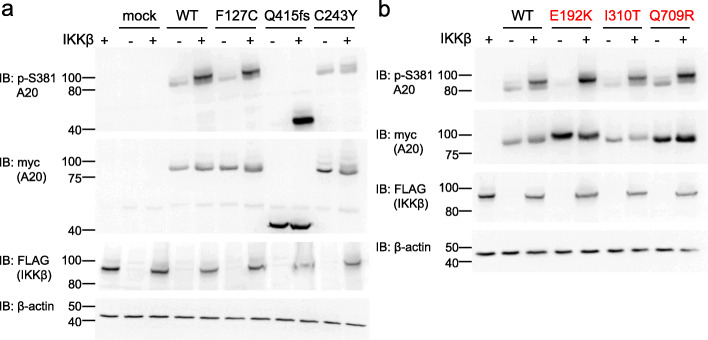
IKKβ-mediated A20 phosphorylation of the *TNFAIP3* variants. Immunoblot of lysates from A20-deficient HEK293 cells transfected with the vectors encoding A20 WT or variants, with or without co-transfected IKKβ, and probed with antibodies to p-S381 A20, myc (detecting A20), FLAG (detecting IKKβ), and β-actin. **a** The loss of IKKβ-mediated S381 phosphorylation was observed with the known mutant C243Y. **b** None of the three variants (E192K, I310T, and Q709R) caused reductions in IKKβ-mediated phosphorylation of A20 S381

A20 Ser381 phosphorylation of the WT was induced by the overexpression of IKKβ and its A20 expression was not reduced, as previously reported [14] (Fig. 4a, b). IKKβ-mediated A20 phosphorylation of known pathogenic mutation Cys243Tyr was found to be reduced compared with that of the WT, while A20 expression of Cys243Tyr was not reduced compared with that without IKKβ transfection. IKKβ-mediated A20 phosphorylation levels of the variants Glu192Lys, Ile310Thr, and Gln709Arg were similar to that of the WT (Fig. 4b).

**Fig. 5:**
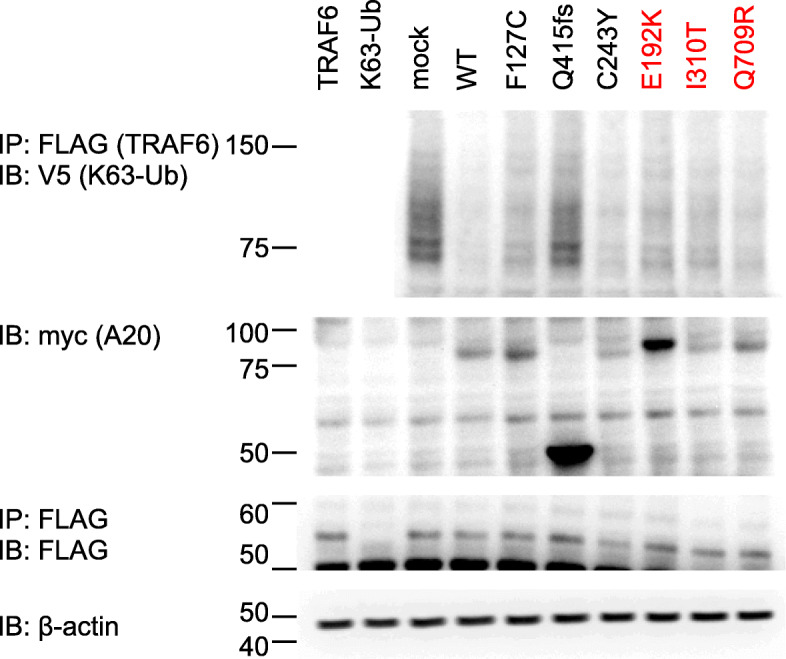
Immunoblot analysis to evaluate the deubiquitinase function of *TNFAIP3* variants*.* A20 WT and variants’ constructs were co-transfected into A20-deficient HEK293 cells with plasmids expressing K63-linked ubiquitin and FLAG-TRAF6, which is the A20 ubiquitination target. Deubiquitinase functions of the variants E192K, I310T, and Q709R did not differ from that of the WT

The formation of K63-polyubiquitin chains for the substrate TRAF6 was detected by immunoprecipitation with anti-FLAG antibody. The *TNFAIP3* mutant Lys417Serfs*4 showed a marked defect in deubiquitination of K63-polyubiquitin chains for TRAF6 compared with that for the WT, as previously reported [1]. The variants Glu192Lys, Ile310Thr, Gln709Arg, and Cys243Tyr did not show differences in deubiquitination from the WT (Fig. 5). Table 2 shows a summary of the three *TNFAIP3* variants and the variant classification based on the results of in vitro functional assay and ACMG standards and guidelines [8].

## Discussion

A20 consists of an amino-terminal ovarian tumor (OTU) domain followed by seven zinc finger (ZnF) domains. It has been reported that each of the domains has the following inhibitory functions on the NF-κB signaling pathway. The OTU domain has deubiquitinating activity that cleaves K63-linked polyubiquitin chains from receptor interacting protein 1 (RIP1), TRAF6, and IKKγ [15–17]. ZnF4 has E3 ligase functions, supporting the K48-linked ubiquitylation and proteasomal degradation of RIP1 [16]. A20 is recruited to the TNF receptor complex by binding ZnF7 and a linear polyubiquitin chain and inhibits the formation of linear ubiquitin chain assembly complex (LUBAC) and IKK complex [18]. A20 also undergoes post-translational modifications; the inhibitory activities of A20 protein were shown to be enhanced by IKKβ-mediated serine phosphorylation near the ZnF domains, notably at S381, and the phosphorylation promotes A20-mediated cleavage of K63-linked polyubiquitin chains [19, 20]. To date, only three missense variants—Cys243Tyr, Met476Ile, and Thr647Pro—have been reported as pathogenic mutations [10–12]. The increase of NF-κB phosphorylated p65 transcription factor in *TNFAIP3* Thr647Pro patients’ lymphocytes and dermal fibroblast cells was identified by flow cytometry, although in vitro assessment was not performed. Moreover, some *TNFAIP3* missense variants identified in tumors, including Met476Ile, showed a significant difference from the WT in the NF-κB reporter gene assay, by co-transfection of the GoF mutation of *CARD11*, which activates BCR signaling [6]. In fact, in this study, not only Cys243Tyr and Thr647Pro but also Met476Ile could not significantly suppress the NF-κB reporter gene activity induced by co-transfection of the GoF mutation of *CARD11*. Thus, this in vitro assay system revealed the pathogenicity of these missense variants. Regarding the evaluation of the NF-κB reporter gene activity of Met476Ile, the method using A20-deficient cells in the present study showed a clearer significant difference from WT than the previously reported similar method with HEK293T cells. Additionally, some missense variants, such as Ser381Ala (substituted with unphosphorylated alanine) and Cys243Tyr, showed a decrease of IKKβ-mediated phosphorylation of A20 and impaired suppression of NF-κB reporter gene activity, which suggested impaired inhibitory function of A20 [14]. On the basis of these previous findings and the results of in vitro assays including for Glu192Lys in this study, it was suggested that the evaluation of NF-κB reporter gene activity by co-transfection of the GoF mutant of *CARD11* using A20-deficient HEK293 cells could more sensitively distinguish missense variants from the WT than other in vitro assays.

To the best of our knowledge, the variant Glu192Lys of the *TNFAIP3* gene has not been previously reported. While in silico analysis predicted that this variant probably damages protein function, our in vitro functional assay revealed the loss of function of this variant. This variant is located in the OTU domain, where many previously reported truncating mutations are located. Although the OTU domain of A20 mediates deubiquitinating activity [21], the variant Glu192Lys and the known pathogenic mutation Cys243Tyr were associated with retention of this activity. The variant Glu192Lys also maintained phosphorylation of A20, but it could not suppress the NF-κB reporter gene activity elevated by TNF-α stimulation or co-transfection of GoF mutants of *MYD88* or *CARD11*. It was suggested that the excessive NF-κB signaling is due to ZnF7 rather than the OTU domain [18, 22]. Thus, it is unclear why this variant and Cys243Tyr located in the OTU domain reduce the inhibitory effect of NF-κB activation, but it might be related to other factors such as changes in the internal domain conformation.

Patient 1 showed a PFAPA-like clinical course with some atypical features but did not fulfill the diagnostic criteria of BD [23]. Recently, the genetic similarities among recurrent aphthous stomatitis, PFAPA, and BD have been demonstrated and it has been proposed that these entities can be grouped together as BD spectrum disorders (BSDs) [24]. Although the *TNFAIP3* gene was not described in that report, HA20 patients also present with early-onset BD-like symptoms, PFAPA, and recurrent stomatitis [5, 25–27], so HA20 might be associated with BSDs. Therefore, patient 1 and his father with Glu192Lys might be classified as having PFAPA or recurrent stomatitis, which present intermediate or mild severity in BSDs.

The variant Ile310Thr of the *TNFAIP3* gene is notably rare in the available databases and the variant Gln709Arg has not been previously reported. These two variants are located in the functional domains of A20, OTU and ZnF6, respectively. However, in the functional analyses, they showed no significant disruption of the inhibitory effect of A20. Several lymphoma-associated missense mutations were reported in ZnF6 [28]. p.Gln709His (genomic mutation ID: COSV52803693), a missense mutation in the same residue of A20, has been registered in the Catalogue of Somatic Mutations in Cancer v92 (COSMIC, https://cancer.sanger.ac.uk/cosmic) and reported as a somatic mutation in adenocarcinoma of the colon. Using our in vitro assay system, these mutations and Gln709del were revealed to be able to suppress NF-κB reporter gene activity (Supplementary Fig. S2). These results suggested that the substitution of residue 709 of A20 might not affect the NF-κB signaling pathway. In this study, although patients 2 and 3 were clinically suggested to have atypical inflammatory diseases, we interpreted Ile310Thr and Gln709Arg as being of uncertain significance and likely benign, respectively, based on ACMG standards and guidelines; this is because the impairment of A20 function was not observed by in vitro functional assays performed in this study and the genetic trait is incomplete between families with the same variant while the allele frequency of the variant was low. The difference of variant classification between Ile310Thr and Gln709Arg was only due to conflicting results of the in silico prediction methods for the variant Ile310Thr in terms of whether it is damaging or benign.

SNPs related to the *TNFAIP3* gene had been well analyzed regarding the association with several autoimmune diseases including SLE [29]. Phe127Cys was the most analyzed one, but no significant disruption of the inhibitory effect of A20 by it could be shown in this study. It is known that, in other autoinflammatory diseases, low-penetrance variants such as *TNFRSF1A*-Arg121Gln (Arg92Gln) and *NLRP3*-GlnQ703Lys, which may not be directly involved in the onset of diseases but can contribute to some extent, have been reported [4]. Although it was difficult to demonstrate the pathological significance by in vitro functional assays, the possibility that *TNFAIP3* Ile310Thr and Gln709Arg are low-penetrance mutations could not be completely ruled out, as with the above-mentioned variants. Assessment of the pathological significance of rare variants has become more important because of the recent progress of comprehensive gene analysis, so methods for functional analysis with higher sensitivity and specificity are needed.

## Conclusions

In this study, we evaluated the pathogenic classification of three novel variants of the *TNFAIP3* gene based on the clinical characteristics of the patients and various in vitro functional assays. In vitro functional analysis showed disruption of the inhibitory effects of A20 by the variant Glu192Lys. To date, genetic analysis with a next-generation sequencer has frequently revealed variants of unknown significance, but the in vitro functional analysis in our study would enable evaluation of the pathological significance of these *TNFAIP3* variants including previously reported mutations. And, in vitro functional analysis is recommended when the identified variant is de novo or the family members with the same *TNFAIP3* variant have symptoms that are consistent with HA20. However, it is necessary to consider the possibility that they are in a pre-disease state or have unnoticed minor symptoms because the clinical features of HA20 have been shown to vary quite markedly.

## Supplementary Information

**Additional file 1.**

## Data Availability

The datasets used and analyzed during the current study are available from the corresponding author upon reasonable request.

## Abbreviations

BCR: B-cell receptor
BD: Behçet’s disease
BSD: Behçet’s disease spectrum disorder
*CARD11*: Caspase recruitment domain family member 11
CRP: C-reactive protein
FMF: Familial Mediterranean fever
HA20: A20 haploinsufficiency
IKKβ: IκB kinase β
OTU domain: Ovarian tumor domain
PFAPA: Periodic fever, aphthous stomatitis, pharyngitis, and adenitis
RIP1: Receptor interacting protein 1
SLE: Systemic lupus erythematosus
TCR: T-cell receptor
TGS: Target gene sequencing
TLR: Toll-like receptor
TNF: Tumor necrosis factor
TRAF6: TNF receptor-associated factor 6
WES: Whole-exome sequencing
ZnF: Zinc finger domain
