# Supplementary material for: Functional analysis of novel A20 variants in patients with atypical inflammatory diseases
Source: Arthritis Res Ther. 2021 Feb 6;23:52. doi: 10.1186/s13075-021-02434-w (PMC7866758; doi:10.1186/s13075-021-02434-w)
Supplement: Supplementary file 1 — Additional file 1. [file 13075_2021_2434_MOESM1_ESM.docx]

**Electronic supplementary materials**

**Functional analysis of novel A20 variants in patients with atypical inflammatory diseases**

Saori Kadowaki, Kunio Hashimoto, Toyoki Nishimura, Kenichi Kashimada, Tomonori Kadowaki, Norio Kawamoto, Kohsuke Imai, Satoshi Okada, Hirokazu Kanegane, Hidenori Ohnishi

**Supplemental Table S1.** Results of the multicolor flow cytometry of the lymphocytes of patients

Patient No. ^a^2-6 years P1 ^a^≧20 years P2 P3

T cells (% of lymphocytes) 69.0±9.0 **42.6 ↓** 67.8±5.4 **80.7 ↑** **60.3 ↓**

Helper T cells (% of CD3) 60.7±7.3 **52.2 ↓** 59.9±9.9 58.3 58.7

Cytotoxic T cells (% of CD3) 29.7±6.7 **40.0 ↑** 34.1±8.7 34.6 36.7

Double negative T cells (% of CD3^+^TCRαβ^+^) 1.34±0.51 0.97 0.77±0.35 **1.17 ↑** **1.31 ↑**

Regulatory T cells (% of CD3^+^CD4^+^) 1.65±0.83 **3.33 ↑** 3.11±1.02 3.94 **4.18 ↑**

Th1 cells (% of CD3^+^CD4^+^CD45RO^+^) 25.0±9.5 9.75 22.6±8.7 15.4 **8.03 ↓**

Th2 cells (% of CD3^+^CD4^+^CD45RO^+^) 41.4±10.6 **60.5 ↑** 35.3±13.8 30.2 **51.0 ↑**

Th17 cells (% of CD3^+^CD4^+^CD45RO^+^) 22.0±6.2 24.0 23.7±4.3 **35.5 ↑** **36.6 ↑**

Follicular helper T cells (% of CD3^+^CD4^+^) 3.26±1.77 **1.13** **↓** 7.02±3.43 **10.60** ↑ **3.40 ↓**

^a^ Normal range at each age in the previous report [13]. If the value deviates from the normal range, it is shown in bold type. Arrows indicate the higher or lower compared to the normal range.

**Supplemental Table S2.** Amino acid alignment of A20 protein amongst representative species around the site of Glu192, Ile310 and Gln709.

| Human protein Index | | 1 | 2 | 3 | 4 | 5 | … | 189 | 190 | 191 | 192 | 193 | 194 | 195 | … |  |  |  |
| --- | --- | --- | --- | --- | --- | --- | --- | --- | --- | --- | --- | --- | --- | --- | --- | --- | --- | --- |
| Homo sapiens | NP_001257437.1 | M | A | E | Q | V |  | N | S | L | E | E | I | H |  |  |  |  |
| Pan troglodytes | XP_003950970.1 | M | A | E | Q | V |  | N | S | L | E | E | I | H |  |  |  |  |
| Rattus norvegicus | XP_001060914.4 | M | A | E | Q | L |  | N | S | L | E | E | I | H |  |  |  |  |
| Mus musculus | NP_001159874.1 | M | A | E | Q | L |  | N | S | L | E | E | I | H |  |  |  |  |
| Bos taurus | NP_001179099.1 | M | A | E | Q | L |  | Q | S | L | E | E | I | H |  |  |  |  |
| Macaca mulatta | NP_001248273.1 | M | A | E | Q | V |  | N | S | L | E | E | I | H |  |  |  |  |
| Gallus gallus | XP_015139672.1 | M | A | G | Q | H |  | N | A | L | E | E | I | H |  |  |  |  |
|  | | | | | | | | | | | | | | | | | | |
| Human protein Index | | 307 | 308 | 309 | 310 | 311 | 312 | 313 | … | 706 | 707 | 708 | 709 | 710 | 711 | 712 | … |  |
| Homo sapiens | NP_001257437.1 | L | M | V | I | E | I | P |  | R | T | T | Q | S | T | S |  |  |
| Pan troglodytes | XP_003950970.1 | L | M | V | I | E | I | P |  | R | T | T | Q | S | T | S |  |  |
| Rattus norvegicus | XP_001060914.4 | L | M | V | L | E | I | P |  | R | T | T | Q | G | A | S |  |  |
| Mus musculus | NP_001159874.1 | L | I | V | M | E | I | P |  | R | T | T | Q | V | A | S |  |  |
| Bos taurus | NP_001179099.1 | L | M | V | V | E | I | P |  | R | A | S | Q | S | A | S |  |  |
| Macaca mulatta | NP_001248273.1 | L | M | V | I | E | I | P |  | R | T | T | Q | S | T | S |  |  |
| Gallus gallus | XP_015139672.1 | L | I | V | I | E | I | P |  | R | A | A | L | T | S | Q |  |  |
|  | | | | | | | | | | | | | | | | | | |
| Human protein Index | | 786 | 787 | 788 | 789 | 790 |  |  | | | | | | | | | | |
| Homo sapiens | NP_001257437.1 | K | Q | M | Y | G |  |  |  |  |  |  |  |  |  |  |  |  |
| Pan troglodytes | XP_003950970.1 | K | Q | M | Y | G |  |  |  |  |  |  |  |  |  |  |  |  |
| Rattus norvegicus | XP_001060914.4 | K | Q | M | Y | G |  |  |  |  |  |  |  |  |  |  |  |  |
| Mus musculus | NP_001159874.1 | K | Q | M | Y | G |  |  |  |  |  |  |  |  |  |  |  |  |
| Bos taurus | NP_001179099.1 | K | Q | M | Y | G |  |  |  |  |  |  |  |  |  |  |  |  |
| Macaca mulatta | NP_001248273.1 | K | Q | M | Y | G |  |  |  |  |  |  |  |  |  |  |  |  |
| Gallus gallus | XP_015139672.1 | K | Q | I | Y | G |  |  |  |  |  |  |  |  |  |  |  |  |

**Supplemental Fig. S1**

CADD vs. minor allele frequency (MAF) plot of *TNFAIP3* by PopViz-2 (http://shiva.rockefeller.edu/PopViz2/). The horizonal axis shows the MAF scores, and the vertical axis shows the CADD scores. The three variants evaluated in this study highlighted in red.

**Supplemental Fig. S2**

NF-κB reporter gene activity of Q709-substituted variants of *TNFAIP3* using A20-deficient HEK293 cells and the expression of A20. **a** NF-κB reporter gene activity was induced by co-transfection with *CARD11* GoF mutant (F130V). The variant Q709R, Q709H and Q709del could suppress the NF-κB reporter gene activity elevated by co-transfection of *CARD11* GoF mutant. **b** Immunoblot analysis of Q709-substituted variants of *TNFAIP3*. The expression level of the three variants were not reduced.
